# Identification of a cytokine network sustaining neutrophil and Th17 activation in untreated early rheumatoid arthritis

**DOI:** 10.1186/ar3168

**Published:** 2010-10-20

**Authors:** Rita Cascão, Rita A Moura, Inês Perpétuo, Helena Canhão, Elsa Vieira-Sousa, Ana F Mourão, Ana M Rodrigues, Joaquim Polido-Pereira, Mário V Queiroz, Henrique S Rosário, Maria M Souto-Carneiro, Luis Graca, João E Fonseca

**Affiliations:** 1 Rheumatology Research Unit, Instituto de Medicina Molecular, Edifício Egas Moniz, Faculdade de Medicina da Universidade de Lisboa, Av. Professor Egas Moniz, Lisboa, 1649-028, Portugal; 2 Rheumatology Department, Centro Hospitalar de Lisboa Norte, EPE, Hospital de Santa Maria, Av. Professor Egas Moniz, Lisboa, 1649-028, Portugal; 3 Rheumatology Department, Centro Hospitalar de Lisboa Ocidental, EPE, Hospital Egas Moniz, Rua da Junqueira, 126, Lisboa,1300, Portugal; 4 Microvascular Biology and Inflammation Unit, Instituto de Medicina Molecular, Edifício Egas Moniz, Faculdade de Medicina da Universidade de Lisboa, Av. Professor Egas Moniz, Lisboa, 1649-028, Portugal; 5 Chronic Inflammation Group, Center for Neurosciences and Cell Biology, Rua Larga 6, Coimbra, 3030, Portugal; 6 Cellular Immunology Unit, Instituto de Medicina Molecular, Edifício Egas Moniz, Faculdade de Medicina da Universidade de Lisboa, Av. Professor Egas Moniz, Lisboa, 1649-028, Portugal

## Abstract

**Introduction:**

Rheumatoid arthritis (RA) is a chronic inflammatory autoimmune disease characterized by sustained synovitis. Recently, several studies have proposed neutrophils and Th17 cells as key players in the onset and perpetuation of this disease. The main goal of this work was to determine whether cytokines driving neutrophil and Th17 activation are dysregulated in very early rheumatoid arthritis patients with less than 6 weeks of disease duration and before treatment (VERA).

**Methods:**

Cytokines related to neutrophil and Th17 activation were quantified in the serum of VERA and established RA patients and compared with other very early arthritis (VEA) and healthy controls. Synovial fluid (SF) from RA and osteoarthritis (OA) patients was also analyzed.

**Results:**

VERA patients had increased serum levels of cytokines promoting Th17 polarization (IL-1β and IL-6), as well as IL-8 and Th17-derived cytokines (IL-17A and IL-22) known to induce neutrophil-mediated inflammation. In established RA this pattern is more evident within the SF. Early treatment with methotrexate or corticosteroids led to clinical improvement but without an impact on the cytokine pattern.

**Conclusions:**

VERA patients already display increased levels of cytokines related with Th17 polarization and neutrophil recruitment and activation, a dysregulation also found in SF of established RA. 0 Thus, our data suggest that a cytokine-milieu favoring Th17 and neutrophil activity is an early event in RA pathogenesis.

## Introduction

Rheumatoid arthritis (RA), the most common chronic autoimmune disease, affects approximately 1% of the population worldwide. This disease comprises a syndrome of pain, stiffness, and symmetrical synovitis which leads to joint destruction, functional disability, and substantial comorbidity due to the involvement of multiple organs and systems. The migration of leukocytes toward the synovium is crucial for the establishment of a chronic inflammatory process in RA [1-3]. This multi-regulated mechanism involves interactions with endothelial cells through cell adhesion molecules and complex cytokine and chemokine pathways.

Neutrophils specifically play an important role in the onset and perpetuation of RA, not only as interleukin (IL)-producing cells but also as cells responsible for the release of high amounts of reactive oxygen species and destructive enzymes, such as metalloproteases, contributing to joint erosions [4]. Neutrophils are among the first leukocytes to arrive at sites of inflammation. In fact, these cells are the most abundant in the synovial fluid (SF) of patients with active RA, and previous results from our group showed that the synovial tissue is heavily infiltrated by neutrophils in the first weeks of RA onset [5]. Interestingly, in animal models of arthritis, neutrophil depletion prevented joint inflammation if neutrophil-depleting antibodies were given before the induction of arthritis. Moreover, when the depleting antibody was given very early after the induction of arthritis, complete abrogation of the inflammatory symptoms was achieved [6].

T helper 17 (Th17) cells have also been proposed to have a relevant role in the early phase of RA through the production of IL-17 [7,8]. This cytokine promotes the recruitment and survival of neutrophils, induces the secretion of proinflammatory cytokines and the upregulation of RANKL (receptor activator of nuclear factor-kappa B ligand), and stimulates the activity of matrix metalloproteases, leading to cartilage catabolism and bone resorption [9,10]. The recruitment, activation, and effector function of Th17 cells and neutrophils are driven by a network of cytokines and chemokines secreted by multiple cellular sources. In established RA, it has been reported that IL-1β, IL-6, IL-8, IL-17, and tumor necrosis factor are elevated in the serum and this correlates with a higher disease activity [11-13]. Nevertheless, our knowledge of the influence of the cytokine network on RA onset remains limited. The characterization of the cytokine profile at this stage, where the transition from an acute to a chronic inflammatory phase occurs, may lead to the identification of early key players, with potential implications for early treatment strategies.

Thus, the main goal of our work was to determine whether cytokines driving neutrophil and Th17 cell activation and proinflammatory function were already present in very early RA (with less than 6 weeks of disease duration) and how this early cytokine environment differs from established RA. We also evaluated whether the introduction of low-dose corticosteroids and methotrexate (MTX) therapy had any influence on the cytokine profile observed at that early stage of the disease. We found that cytokines related to Th17 polarization and neutrophil recruitment and activation were elevated in early RA and that the conventional therapeutic options, though able to control clinical manifestations of the disease, were ineffective in reversing this underlying proinflammatory drive.

## Materials and methods

### Patients

Blood samples were obtained from 38 consecutive untreated polyarthritis patients with less than 6 weeks of disease duration. Some of these patients (19), after a minimum follow-up of 3 months, fulfilled the 1987 American College of Rheumatology (ACR) criteria for RA [14]. These patients were classified as very early rheumatoid arthritis (VERA) patients, and further samples were collected 4 to 6 weeks after starting a low dose of oral corticosteroids (5 to 10 mg of prednisone) (time 1) and 4 months after reaching the minimum effective dose of MTX (time 2) (up to a maximum of 20 mg/week) that was required to reduce the disease activity score using 28 joint counts (DAS28) to less than 3.2 [15]. The remaining early arthritis patients (19), who did not develop RA, were classified as very early arthritis (VEA). Baseline blood samples from VERA and VEA patients were compared with 27 healthy donors used as controls. Additionally, 12 blood and 15 SF samples were obtained from patients with established RA. SF samples were also collected from 10 patients with osteoarthritis (OA) (Rheumatology Department, Hospital de Santa Maria, Lisbon, Portugal) (Table 1). Owing to the clinical characteristics of the VEA patients, SF in easily accessible joints was not available in VERA and VEA patients and thus SF was not analyzed in these groups of patients. The health assessment questionnaire (HAQ) [16] and DAS28 were applied to all patients. The study was approved by the local ethics committee, and all patients signed an informed consent form. Patient care was conducted in accordance with standard clinical practice, and the study was performed in accordance with the Declaration of Helsinki as amended in Edinburgh (2000).

**Table 1:** Clinical information about healthy controls and patients with VERA, VEA, RA, or OA

	Controls (*n *= 24)	VERA (*n *= 19)			VEA (*n *= 19)	RA (*n *= 12)	RA SF (*n *= 15)	OA SF (*n *= 10)
						
		Baseline	Time 1	Time 2				
Age in years, mean ± SD	40 ± 13	50 ± 17			40 ± 13	63 ± 10	57 ± 10	67 ± 13
Sex, female/male	17/7	16/3			15/4	11/1	11/4	5/5
DAS28, mean ± SD	NA	6.1 ± 1.8	4.1 ± 1.6^a^	3.1 ± 1.6^a^	4.5 ± 1.6^a^	5.2 ± 1.0	4.6 ± 1.4	NA
HAQ, mean ± SD	NA	1.4 ± 0.8	0.8 ± 0.7^a^	0.8 ± 0.7	0.8 ± 0.6^a^	1.5 ± 1.0	1.4 ± 0.8	NA
RF-positive, %	ND	42	ND	ND	0	67	ND	ND
Anti-CCP-positive, %	ND	32	ND	ND	0	45	ND	ND

^a^Disease activity score using 28 joint counts (DAS28) and health assessment questionnaire (HAQ) values were compared between very early rheumatoid arthritis (VERA) and very early arthritis (VEA) patients with reference to VERA baseline values. Differences were considered statistically significant for *P *values of less than 0.05. anti-CCP, anti-cyclic citrullinated peptide; IL, interleukin; MTX, methotrexate; NA, not applicable; ND, not determined; OA, osteoarthritis; RA, rheumatoid arthritis; RF, rheumatoid factor; SD, standard deviation; SF, synovial fluid.

### Cytokine quantification

IL-1β, IL-2, IL-4, IL-6, IL-8, IL-10, IL-12(p70), IL-17A, IL-22, IL-23, and interferon-gamma levels were measured in the serum and SF by FlowCytomix assay kit (Bender MedSystems, Vienna, Austria) in accordance with the instructions of the manufacturer. Standard curves for each cytokine were generated by using reference cytokine concentrations supplied by the manufacturer. Samples were acquired with a FACS Calibur flow cytometer (BD Biosciences, San Jose, CA, USA). Raw data of the flow cytometry bead assay were analyzed by FlowCytomix Pro 2.2 software (Bender MedSystems).

### Measurement of autoantibodies

Rheumatoid factor (RF)-IgM was determined in all patients by means of an IMTEC Autoimmune Diagnostics ELISA [enzyme-linked immunosorbent assay] kit (Human GmbH, Wiesbaden, Germany) in accordance with instructions of the manufacturer, and samples were processed using a ChemWell 2910 automated analyzer (GMI, Ramsey, Minnesota, USA). Serum levels of anti-cyclic citrullinated peptide (anti-CCP) were measured by ELIA™ CCP test system (Phadia GmbH, Freiburg, Germany), and samples were analyzed with an ImmunoCAP 100 instrument (Phadia GmbH).

### Statistical analysis

Statistical differences were determined with non-parametric Kruskal-Wallis, Mann-Whitney, and Wilcoxon signed-rank tests and GraphPad Prism (GraphPad Software, Inc., San Diego, CA, USA). Correlation analysis was performed with the Spearman test. Differences were considered statistically significant for *P *values of less than 0.05.

## Results

### Characterization of patients and disease evaluation

A total of 38 polyarthritis patients with less than 6 weeks of disease duration were evaluated. Nineteen patients fulfilled the 1987 ACR criteria for RA after a minimum follow-up of 3 months and were classified as VERA patients. The mean age of the VERA patients was 59 ± 17 years, 84% were female, 42% were RF-positive and 32% anti-CCP-positive, the initial DAS28 was 6.1 ± 1.8, and the initial HAQ was 1.4 ± 0.8. After treatment with low doses of prednisone and MTX, there was a significant reduction of both DAS28 and HAQ values (Table 1). The group of VEA patients included 19 patients, and 14 of them later had one of the following diagnoses: spondylarthritis (5 cases), systemic lupus erythematosus (4 cases), crystal induced arthritis (2 cases), Sjögren syndrome (1 case), paraneoplastic polyarthritis related to multiple myeloma (1 case), and arthritis associated with HIV infection (1 case). Five patients entered spontaneously into remission before 3 months of follow-up, remaining without a specific diagnosis and were thus classified as presenting a self-limited form of polyarthritis. The mean age of the VEA patients was 40 ± 13 years, 79% were female, all patients were RF-negative and anti-CCP-negative, the initial DAS28 was 4.5 ± 1.6, and the initial HAQ was 0.8 ± 0.6. Both DAS28 and HAQ values were significantly lower than those of VERA patients at baseline (Table 1). These early polyarthritis patients represent a subset of a larger cohort previously described by our group [17].

Furthermore, blood samples were collected from 12 patients with established RA; mean age was 60 ± 10 years, 92% were female, and 67% were RF-positive and 45% anti-CCP-positive (Table 1). Additionally, SF samples were collected from 12 patients with established RA; mean age was 57 ± 10 years, and 73% were female (Table 1). The established RA group of patients had a DAS28 and a HAQ mean scores similar to VERA baseline values.

### IL-8 is increased in VERA patients and locally in the joints of patients with established RA

**Figure 1:**
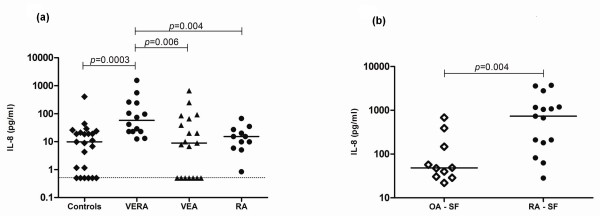
**Interleukin-8 (IL-8) is increased in the serum of very early rheumatoid arthritis (VERA) patients and in synovial fluid (SF) of established rheumatoid arthritis (RA)**. **(a) **The serum concentration of IL-8 was measured in VERA and very early arthritis (VEA) patients as well as healthy controls and patients with established RA. The serum concentration of IL-8 was increased in VERA patients compared with any other group. Dotted line represents the limit of detection for the assay. **(b) **The concentration of IL-8 was measured in the SF collected from patients with established RA and from a control group with osteoarthritis (OA). We found a significant increase of IL-8 in RA-SF. Differences were considered statistically significant for *P *values of less than 0.05 according to the Mann-Whitney test.

Given the proposed role of neutrophils in the pathogenesis of RA [18,19], we quantified the major neutrophil chemoattractant, IL-8, in the serum of VERA patients. At baseline, VERA patients had significantly higher levels of IL-8 when compared with both VEA and healthy controls (Figure 1a). After 2 to 4 weeks of low-dose corticosteroids and after 4 months of MTX therapy, there were no significant changes in the levels of circulating IL-8 (data not shown). Interestingly, VERA patients also had significantly higher circulating levels of IL-8 in comparison with serum from established RA (Figure 1a). Neutrophils accumulate locally in the joints of patients with RA [20]. Thus, we quantified the concentration of IL-8 in the SF of patients with RA and compared the concentration with that of SF from patients with OA. We found significantly higher levels of IL-8 in the SF of patients with RA in comparison with OA SF (Figure 1b).

### IL-17 levels are dysregulated in both VERA patients and patients with established RA

**Figure 2:**
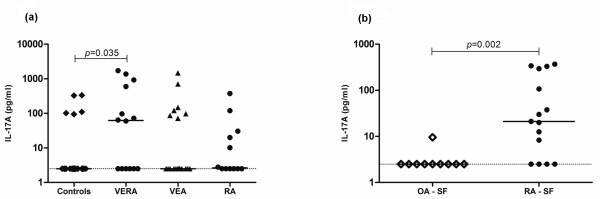
**Very early rheumatoid arthritis (VERA) patients and synovial fluid (SF) of established rheumatoid arthritis (RA) display increased levels of interleukin-17A (IL-17A)**. **(a) **The serum concentration of IL-17A was measured in VERA and very early arthritis (VEA) patients as well as healthy controls and patients with established RA. The serum concentration of IL-17A was increased in VERA patients compared with healthy controls. **(b) **The concentration of IL-17A was measured in the SF collected from patients with established RA and from a control group with osteoarthritis (OA). In the SF of patients with RA, we observed a significant increase of IL-17A. Dotted lines represent the limit of detection for the assay. Differences were considered statistically significant for *P *values of less than 0.05 according to the Mann-Whitney test.

Previous studies from our group showed that there is a delay in the apoptosis of circulating neutrophils in VERA patients [21]. Therefore, we analyzed IL-17A levels in these patients since it has already been described that this cytokine is important for the survival of neutrophils [22]. Moreover, IL-17A is a signature cytokine of Th17 cells, a subset proposed to have a key role in RA pathogenesis [9,23]. We found that VERA patients had significantly higher levels of IL-17A when compared with healthy controls, but not with VEA patients (Figure 2a). Furthermore, in our previous work, we found no difference in the frequency and absolute numbers of CD4^+ ^and CD8^+ ^T-cell subpopulations in the peripheral blood of these patients when analyzed by flow cytometry [17].

Regarding the effects of early therapy, we found that neither corticosteroids nor MTX affected the level of IL-17A (data not shown). Moreover, IL-17A was significantly increased locally within the joints of patients with established RA in comparison with control SF from patients with OA (Figure 2b).

### RA has a Th17-cytokine pattern since the very first weeks of onset

**Figure 3:**
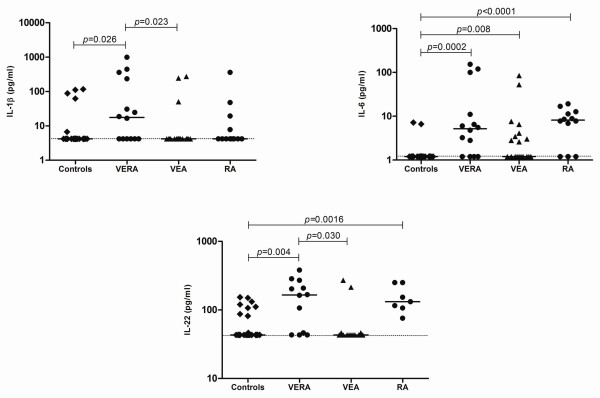
**Cytokines related to T helper 17 (Th17) polarization are increased in the serum of very early rheumatoid arthritis (VERA) patients and synovial fluid of established rheumatoid arthritis (RA)**. The serum concentrations of interleukin (IL)-1β, IL-6, and IL-22 were measured in VERA and very early arthritis (VEA) patients as well as healthy controls and patients with established RA. All three cytokines were increased in VERA patients compared with healthy controls. IL-6 was equally elevated in all groups of patients with an inflammatory disease, whereas the other two cytokines were increased only in VERA (IL-1β) or in VERA and RA patients (IL-22). Dotted lines represent the limit of detection for the assays. Differences were considered statistically significant for *P *values of less than 0.05 according to the Mann-Whitney test.

Having found that IL-17A was elevated in VERA patients, we decided to quantify a panel of cytokines known to be associated with Th17 polarization. At baseline, VERA patients had significantly higher levels of IL-1β and IL-22 in comparison with both VEA and healthy controls. In addition, we found that VERA patients have significantly higher IL-6 levels than healthy controls (Figure 3). Furthermore, the significantly higher circulating levels of IL-6 and IL-22 were maintained in established RA (Figure 3).

**Figure 4:**
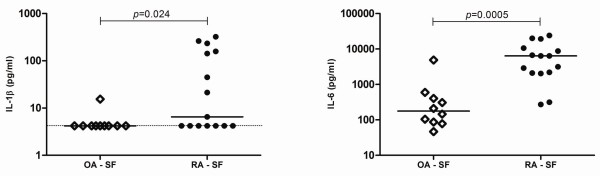
**Cytokines related to T helper 17 (Th17) polarization are increased in the synovial fluid (SF) of established rheumatoid arthritis (RA)**. The concentrations of interleukin (IL)-1β and IL-6 were markedly increased in the SF collected from patients with established RA when compared with osteoarthritis (OA). Dotted lines represent the limit of detection for the assays. Differences were considered statistically significant for *P *values of less than 0.05 according to the Mann-Whitney test.

Locally, within the joints of patients with RA, the SF displayed elevated levels of IL-1β and IL-6 in comparison with OA SF (Figure 4 and Table 2). Moreover, no significant differences could be observed for IL-23 in circulation or locally in the joints (data not shown). We have also studied cytokines associated with the function of Th2 (IL-4 and IL-10) and Th1 (IL-2, IL-12 (p70), and INFγ) cells. However, no statistically significant differences could be observed for any of these cytokines (data not shown).

**Table 2:** Cytokine levels in healthy controls and patients with VERA, VEA, established RA, or OA

Cytokine, pg/mL	Controls	VERA	VEA	RA	RA SF	OA SF
IL-1β	4.2 (4.2-116.8)	17.7 (4.2-99.7)	4.2 (4.2-272.7)	4.2 (4.2-360.3)	6.5 (4.2-322.1)	4.2 (4.2-15.5)
IL-6	1.2 (1.2-7.2)	5.2 (1.2-153.2)	1.2 (1.2-84.7)	8.2 (1.2-19.7)	6361.0 (272.7-24,135.0)	177.6 (46.6-4,881.0)
IL-8	9.9 (0.5-407.7)	57.6 (12.5-1,546.0)	8.9 (0.5-665.2)	15.2 (0.8-67.5)	735.7 (28.3-3,717.0)	48.2 (22.0-680.0)
IL-17A	2.5 (2.5-333.7)	62.0 (2.5-1,714.0)	2.5 (2.5-1,477.0)	2.6 (2.5-375.6)	21.1 (2.5-369.1)	2.5 (2.5-9.5)
IL-22	43.3 (43.3-153.4)	165.3 (43.3-380.6)	43.3 (43.3-270.5)	131.7 (75.8-250.7)	153.4 (75.8-336.0)	151.7 (92.4-235.3)

Values are presented as median (range). IL, interleukin; OA, osteoarthritis; RA, rheumatoid arthritis; SF, synovial fluid; VEA, very early arthritis; VERA, very early rheumatoid arthritis.

## Discussion

Several studies have previously demonstrated that neutrophils play an important role in the onset of RA [21]. This hypothesis is supported by data from animal models [24]. In fact, neutrophils are the most abundant leukocytes in the SF of patients with active RA, and in early RA, these cells show significantly lower levels of apoptosis when compared with patients with other persistent forms of arthritis or with arthritis that has a self-limited disease course [25]. Additionally, previous results from our group demonstrated that there is a delay in the apoptosis of circulating neutrophils in VERA patients [21] and that these cells heavily infiltrate the synovial tissue during RA onset [5].

In the present study, we demonstrate that a neutrophil- and Th17-driving cytokine pattern is present in untreated VERA patients with less than 6 weeks of disease duration. We consider this observation of interest because the knowledge concerning the immune mechanisms associated with the onset of RA is still elusive. In fact, the majority of early RA studies include patients with 3 to 12 months of disease duration or even more. In accordance with an early participation of neutrophils in RA, our results revealed that VERA patients have increased levels of IL-8 when compared with both VEA and healthy controls, and this could explain the preactivated state of circulating neutrophils [18] and their recruitment toward the SF from the very first weeks of RA onset.

In addition, Th17 cells are known to be important for the promotion of neutrophil-mediated inflammation by producing IL-17A, a cytokine known to indirectly activate neutrophil chemotaxis and extend their survival [10,22]. We found a high serum concentration of IL-17A in VERA patients as well as locally within the joints of patients with established RA. This might indicate that an activation of Th17 cells from a very early phase of the disease can promote neutrophil participation in RA pathogenesis [22]. However, we found no evidence for changes in the frequency of T-cell subsets in the peripheral blood of VERA patients [17]. This observation is not unexpected; the relatively small representation of antigen-specific T cells in the circulating pool are the activated T cells that drive the pathology more likely found within the tissues [26]. In a study performed by Kokkonen and colleagues [27], the levels of several cytokines and chemokines were analyzed in blood samples from a group of individuals 3.3 years before RA onset ('pre-patients') and compared with healthy donors and RA patients with 7.7 ± 3.6 months of disease duration. An interesting finding was that IL-17 was present at its highest concentration in pre-patients and the level of this cytokine was lower in patients with RA. This is in accordance with our own results; we observed an increased level of IL-17 in RA patients with less than 6 weeks of disease duration, whereas in patients with established RA, the levels were not significantly different from those of healthy controls. Remarkably, the IL-17 median concentration observed in our established RA cohort (2.6 pg/mL) was even lower than that of RA patients from the work of Kokkonen and colleagues [27] (6.0 pg/mL). Thus, this observation reinforces the role of IL-17 in the initial phase of RA, and as the pathogenesis progresses to a chronic stage, other factors are subsequently brought into action in the peripheral blood. Unlike Kokkonen and colleagues, we have not detected differences in Th1- and Th2-related cytokines between both VERA and patients with established RA in comparison with controls. These discrepancies might be related to the different methodologies used.

Additionally, the elevated levels of IL-1β observed in VERA patients can stimulate endothelial cells, T and B cells, and fibroblasts in the joints to produce IL-6 and IL-8. But importantly, IL-1β and IL-6, both found to be increased in VERA patients, are known to promote the differentiation of Th17 cells, which in turn secrete IL-17A and IL-22 [28,29], two cytokines that were elevated in VERA patients and have an essential function in the pathogenesis of autoimmune diseases [29].

Currently, the treatment of choice for RA at the time of presentation is MTX. Interestingly, in spite of clinical improvement (DAS28 reduced from 6.1 ± 1.8 to 3.1 ± 1.6), neither therapy with low-dose corticosteroids nor combined therapy with low-dose corticosteroids and MTX corrected the dysregulated cytokine pattern observed in VERA patients. In fact, low-dose corticosteroids and MTX have unclear effects on the RA cytokine network. For instance, corticosteroids fail to reduce serum levels of IL-1β and IL-8 [30] and MTX does not alter serum IL-1β concentration when compared with pre-treatment levels [31,32]. Our results suggest that the conditions contributing to Th17 cells and neutrophil-mediated inflammation, thus driving early pathogenesis, are not modified with early treatment with low-dose corticosteroids and MTX.

The elevated IL-1β, IL-6, IL-8, and IL-17A levels observed in the SF of patients with RA confirm a local role for these cytokines in the maintenance of synovitis. Moreover, IL-6 can support a continuous recruitment of autoreactive B cells toward the synovium [33,34], contributing to an exacerbation of the inflammatory process because of the production of autoantibodies and immune complexes.

## Conclusions

Taken together, our data reinforce the potential relevance of therapies targeting IL-1β [35,36] and IL-6 [37,38] in early RA. In addition, the data establish IL-8 and IL-17A as other potential therapeutic targets at an early stage of the disease. Finally, we found that MTX and corticosteroids, though effective in reducing disease activity in VERA patients, do not appear to correct underlying cytokine dysregulation driving the Th17/neutrophil-mediated inflammation.

## Abbreviations

ACR: American College of Rheumatology; anti-CCP: anti-cyclic citrullinated peptide; DAS28: disease activity score using 28 joint counts; HAQ: health assessment questionnaire; IL: interleukin; MTX: methotrexate; OA: osteoarthritis; RA: rheumatoid arthritis; RF: rheumatoid factor; SF: synovial fluid; Th17: T helper 17; VEA: very early arthritis; VERA: very early rheumatoid arthritis.

## Competing interests

The authors declare that they have no competing interests.

## Authors' contributions

RC and RAM equally performed all of the laboratorial work, data collection, and statistical analysis and wrote the paper. IP contributed to some of the laboratory experiments. HC, ES, AFM, AMR, and JP-P were responsible for the selection, follow-up, and medical care of patients enrolled in this study and helped review the paper. MVQ participated as the head of the Rheumatology Department of Hospital de Santa Maria, which approved the study and patients' management. HSR and MMS-C made a substantial intellectual contribution to the present work and revised it critically. LG and JEF, as senior authors, conceived of the study, participated in its design and coordination, and contributed important intellectual input to the draft of the manuscript. All authors read and approved the final manuscript.
